# Promoting patient engagement in cancer genomics research programs: An environmental scan

**DOI:** 10.3389/fgene.2023.1053613

**Published:** 2023-01-18

**Authors:** Anne L. R. Schuster, Norah L. Crossnohere, Jonathan Paskett, Neena Thomas, Heather Hampel, Qin Ma, Jessica C. Tiner, Electra D. Paskett, John F. P. Bridges

**Affiliations:** ^1^ Department of Biomedical Informatics, The Ohio State University College of Medicine, Columbus, OH, United States; ^2^ Division of Clinical Cancer Genomics, City of Hope National Medical Center, Duarte, CA, United States; ^3^ Division of Human Genetics, The Ohio State University Wexner Medical Center, Columbus, OH, United States; ^4^ Epidemiology and Genomics Research Program, Division of Cancer Control and Population Sciences, National Cancer Institute, Bethesda, MD, United States; ^5^ Division of Cancer Prevention and Control, Department of Internal Medicine, College of Medicine, The Ohio State University, Columbus, OH, United States; ^6^ Division of Epidemiology, College of Public Health, The Ohio State University, Columbus, OH, United States

**Keywords:** Cancer Moonshot, patient engagement, patient participation, health equity, genomics

## Abstract

**Background:** A national priority in the United States is to promote patient engagement in cancer genomics research, especially among diverse and understudied populations. Several cancer genomics research programs have emerged to accomplish this priority, yet questions remain about the meaning and methods of patient engagement. This study explored how cancer genomics research programs define engagement and what strategies they use to engage patients across stages in the conduct of research.

**Methods:** An environmental scan was conducted of cancer genomics research programs focused on patient engagement. Research programs were identified and characterized using materials identified from publicly available sources (e.g., websites), a targeted literature review, and interviews with key informants. Descriptive information about the programs and their definitions of engagement, were synthesized using thematic analysis. The engagement strategies were synthesized and mapped to different stages in the conduct of research, including recruitment, consent, data collection, sharing results, and retention.

**Results:** Ten research programs were identified, examples of which include the Cancer Moonshot Biobank, the MyPART Network, NCI-CONNECT, and the Participant Engagement and Cancer Genome Sequencing (PE-CGS) Network. All programs aimed to include understudied or underrepresented populations. Based on publicly available information, four programs explicitly defined engagement. These definitions similarly characterized engagement as being interpersonal, reciprocal, and continuous. Five general strategies of engagement were identified across the programs: 1) digital (such as websites) and 2) non-digital communications (such as radio broadcasts, or printed brochures); 3) partnering with community organizations; 4) providing incentives; and 5) affiliating with non-academic medical centers. Digital communications were the only strategy used across all stages of the conduct of research. Programs tailored these strategies to their study goals, including overcoming barriers to research participation among diverse populations.

**Conclusion:** Programs studying cancer genomics are deeply committed to increasing research participation among diverse populations through patient engagement. Yet, the field needs to reach a consensus on the meaning of patient engagement, develop a taxonomy of patient engagement measures in cancer genomics research, and identify optimal strategies to engage patients in cancer genomics. Addressing these needs could enable patient engagement to fulfill its potential and accelerate the pace of cancer genomic discoveries.

## 1 Introduction

Rapid improvements in genome sequencing technology have revolutionized our ability to molecularly characterize cancer tumors and have, in turn, transformed our understanding of cancer biology (Wang et al., 2016; Wang et al., 2020). The understanding of cancer biology holds promise for improving the diagnosis and treatment of cancer (Hutter and Zenklusen, 2018), but that promise has yet to be realized for many patient populations who have not been adequately included in research. Hundreds of different cancers lack the molecular characterization of tumors required to guide patient therapy (National Cancer Institute, 2016). Similarly, past cancer genomics research inadequately include patients from underrepresented racial and ethnic minority groups and other underserved populations (Spratt et al., 2016). Evidence indicates that large genomic sequencing efforts such as The Cancer Genome Atlas overrepresent non-Hispanic white patients compared to the United States population and underrepresent patients from diverse racial and ethnic backgrounds, especially Asian and Hispanic patients (Spratt et al., 2016; Stein et al., 2021). These shortcomings result in inequities in the development of cancer therapies and the receipt of cancer care.

Several factors contribute to the inadequate characterization of all cancers and the underrepresentation of diverse patient populations in cancer genomics research. Logistical barriers impacting both patients and researchers can hinder access to research (Seruga et al., 2014). Rare cancers are difficult to study due to low disease incidence and patients’ receipt of treatment at geographically dispersed institutions, including community hospitals (Painter et al., 2020). Patients can face additional logistical barriers such as language differences, competing demands for time, and limited access to transportation (George et al., 2014; Seruga et al., 2014). Studying genetics and genomics can add further complications due to information complexity, familial and community relationships, and education needs regarding cancer progression as well as genomics (Rebbeck et al., 2022). Many structural and historical barriers also prevent underrepresented groups from participating in research. Willingness to participate can be dampened by experiences of injustices in the healthcare system, poor communication with researchers or healthcare providers, and distrust based on legacies of exploitive research (Gamble, 1997; Corbie-Smith et al., 2002; Woodahl et al., 2014; Sutton et al., 2019; Geneviève et al., 2020). More specifically, concerns about privacy, unauthorized use of biospecimens, and stigmatizing interpretations of research results can pose barriers to participating in genetics research. These types of misgivings are exemplified by two cases that involved the unauthorized use of samples from the Havasupai in Arizona (Arizona, United States) (Bommersbach, 2008) and the Nuu-Chah-Nulth in British Columbia (Canada), and resulted in a resolution of the National Congress of American Indians affirming tribal ownership of health-related data (National Congress of American Indians, 2022).

In recent years patient engagement has received increasing attention for its potential to democratize the research process and improve the value of research (Cancer Research UK; Deverka et al., 2018; Freeman-Daily et al., 2018; Anampa-Guzmán et al., 2022). Across the world prominent initiatives and national-level infrastructure supports patient engagement in research broadly and genomics research specifically (Cancer Research UK; Candadian Institutes of Health Research; Patient Centered Outcomes Research Institute (PCORI); Patient Focused Medicine; Lough, 2015; I.H.R., 2020; Health, 2022). Despite the attention given to patient engagement in research, the meaning of the term varies, especially across international settings (Cancer Research UK; Murphy et al., 2021; Schuster et al., 2022a). In the United States, the term patient engagement in research has been used to characterize patients’ contributions to research *via* roles that range from “passive” study participants to “active” patients involved in all phases of the research process (Domecq et al., 2014; Katz and Paskett, 2015; Shippee et al., 2015; Fergusson et al., 2018; Hemphill et al., 2020; Rebbeck et al., 2022), which have been previously defined as preparatory, execution, and translational phases of research. Recent work suggests ways to broaden the conceptualization of engaging patients in cancer research in both passive and active roles (Schuster et al., 2022b).

A prominent national initiative in the United States—the Cancer Moonshot^SM^–funds a network of research programs for direct patient engagement (National Cancer Institute, 2016). With funding from the United States National Cancer Institute (NCI), this network aims to directly engage patients to contribute their comprehensive tumor profile data to expand knowledge about what therapies work, in whom, and in which types of cancer (National Cancer Institute, 2022b). The meaning of the term “direct patient engagement” is not defined explicitly, but implicitly entails increasing research participation, especially among understudied and historically underrepresented populations (National Cancer Institute, 2016; 2022b). The purpose of engaging a greater diversity of study participants is to ensure that research and clinical trials can benefit people from all communities. There is, however, a lack of knowledge about how research programs studying cancer genomics operationalize engagement of patients as study participants.

This study sought to: identify relevant cancer genomics research programs in the United States that are focused on increasing the diversity of study participants; describe how they define patient engagement; and characterize the strategies they use to engage study participants in cancer genomics research. Additionally, we sought to identify future directions for research on patient engagement in cancer genomics research. We expect the findings will be of interest to researchers seeking to understand the status of patient engagement in research programs studying cancer genomics. It is also likely to be of interest to researchers seeking more information on the conduct of patient engagement and to funders seeking to advance the science of patient engagement.

## 2 Methods and materials

An environmental scan was conducted (Rowel et al., 2005; Krok-Schoen et al., 2019) of research programs engaging patients in cancer genomics research. For the purposes of this paper, research programs can include research networks comprised of multiple institutions as well as large, centralized genomics data collection initiatives. Environmental scans can describe the context in which decisions and processes are currently and have historically been made. They are an adaptive approach to identifying and synthesizing large and diverse forms of information (Rowel et al., 2005; Krok-Schoen et al., 2019). Additionally, they can inform strategic planning, decision making, and future areas of inquiry (Wilburn et al., 2016). An environmental scan is well-suited to characterize patient engagement in research programs studying cancer genomics given the nascency of the field and the potential lack of information in any one data source.

Research programs were identified *via* publicly available information, a targeted literature review, and interviews with key informants. Research programs were included in the environmental scan if they made explicit reference to engaging patients as study participants in cancer genomics research; were focused on enrolling patients from the United States; and expressed interest in expanding the inclusion of study participants from a diversity of populations across the United States. Programs were excluded if they were solely focused on engaging patients as active contributors to the research process, including the preparation, execution, and translation of research.

Publicly available materials such as program websites, research protocols, abstracts, and grey literature were identified *via* Google searches, forward/backward referencing, and clinicaltrial.gov. Descriptive data were extracted for each program using *a priori* developed tools on: research program description (e.g., target cancer types, target population, enrollment start date(s), study design(s), and key goals); definition of engagement; and strategies for engagement. Descriptive data was also extracted from program websites using *a priori* identified categories related to content, visual design, user engagement principles (Beaunoyer et al., 2017; Mac et al., 2020; W3 Web Accessibility Iniative, 2022) and the strategies used to engage study participants and the stage of research that the strategy corresponds to (e.g., recruitment, consent, data collection, sharing results, retention) (Rebbeck et al., 2022).

The targeted literature review was conducted by searching databases including PubMed, Google Scholar, Embase, and Web of Science. The search strategy for all databases included three general concepts: 1) patient engagement, 2) cancer, and 3) genomics. Search strategies were tailored to databases using appropriate terminology, truncations, and operators. Information was limited to English-language and the years 2010 to present (2022). Articles were selected if they pertained to research programs using patient engagement to study cancer genomics. Data were extracted following an *a priori* defined extraction template that included documenting information such as background information (e.g., title, year); engagement definitions; and the strategies used to engage study participants and the corresponding study activities (Rebbeck et al., 2022).

Interviews were conducted with 18 key informants, who were identified purposively through publicly available information; word-of-mouth; and snowball sampling, whereby key informants directly referred study team members to new potential key informants. Two key informants per included research program were invited to participate in the interviews. Key informants from all but one research program participated in the interviews. Interviews were completed *via* Zoom following a semi-structured interview guide with questions on topics such as the program’s: conceptualizations of engagement, types of engagement strategies used, and perceived strengths or limitations of engagement strategies. These questions directly pertained to the aim of this environmental scan and were intended to accompany and contextualize findings from the other sources of information. Information was recorded using a combination of video recordings, transcriptions, and field notes. On average, the interviews lasted 65 min.

Thematic analysis was used to narratively synthesize the research programs’ written definitions of engagement and to categorize their engagement strategies. The categories of patient engagement strategies for each program were then mapped to different study activities in the conduct of cancer genomics research, including recruitment, consent, data collection, sharing results, and retention. Brief case examples were gathered to illustrate the use of each strategy. This environmental scan was deemed non-human subject research by the Ohio State University College of Medicine Institutional Review Board (OSU IRB #2021E0565).

## 3 Results

In total ten research programs were identified that are engaging patients in cancer genomics research. Table 1 describes the goals and enrollment populations and settings of each research program. The estimated cohort size for studies conducted by each research program ranges from the hundreds to over one million. The cohorts represent a diversity of populations that include individuals across age groups, racial and ethnic identities, geographic locations, and with different types of cancers. Four of the ten programs are funded through the Cancer Moonshot initiative to “Establish A Network for Direct Patient Engagement” (National Cancer Institute, 2022b). They focus on cancers among populations whose data on cancer health risks and outcomes are currently limited, including cancers that are rare; early onset; highly lethal; locally advanced or metastatic; or have high disparities in incidence/mortality. Two of these four programs are also focused on enrolling participants who are medically underserved and/or from diverse geographic and racial and ethnic backgrounds.

**TABLE 1:** Research programs and key information about their populations, protocols, and goals.

Program name	Enrollment population	Diversity focus	Enrollment setting(s)	Key goals
Programs funded *via* Cancer Moonshot’s initiative for direct patient engagement				
Cancer Moonshot Biobank (National Cancer Institute, 2022a)	Adolescents or adults diagnosed with 1 of 7 locally advanced or metastatic cancers	Rural, medically underserved; racial and ethnic minorities	NCORP	Accelerate research on drug resistance and sensitivity
MyPART Network (National Cancer Institute, 2022e)	Children, teens, and young adults with solid rare tumors	Not a specific focus	Remote; NIH Clinical Center	Accelerate treatment discovery for tumors without cures
NCI-CONNECT (National Cancer Institute, 2022f)	Adults with 12 rare central nervous system cancers	Not a specific focus	Remote; NIH Clinical Center	Improve understanding, standards of care, and patient outcomes
PE-CGS Network (National Cancer Institute, 2022g)	Children and adults with rare, highly lethal, early onset, high disparities in incidence and/or mortality, or cancers in understudied populations	Rural and medically underserved, low literacy, racial and ethnic minorities	Remote; Academic and non-academic medical centers	Generate new discoveries and transform engagement in cancer genomics
Programs external to the Cancer Moonshot’s initiative for direct patient engagement				
All of Us Research Program (All of Us Research Program, 2021)	All adults (healthy or with any disease)	Persons underrepresented in biomedical research	Remote; Academic and non-academic medical centers	Enable a new era of medicine through research, policies, and technology
Connect for Cancer Prevention Study (National Cancer Institute, 2022d)	Adults aged 40–65 without a history of cancer	Racial and ethnic minorities; rural, and medically underserved	Academic and non-academic medical centers	Improve understanding of cancer causes and prevention
CCDI (National Cancer Institute, 2022c)	Children, adolescents and young adults with any cancer	All children with cancer	Academic medical center	Speed diagnosis and inform treatment
CSER II^a^(CSER, 2022a)	Children with cancer; adults at risk for hereditary cancer	Racial and ethnic minorities, low SES medically underserved	Academic and non-academic medical centers; outpatient clinic	Understand integration of genomics in clinical care of diverse individuals
Count Me In(Count Me In, 2022)	Children, adults with any cancer	Persons with rare cancer	Remote	Accelerate biomedical research through direct patient engagement
eMERGE Network (eMERGE Network, 2022)	Children, adults with cancers represented in patient population	Persons of diverse ancestry	Academic medical centers	Combine biobanks with EMRs for large scale genetic research

^a^ Reporting on two (of seven) CSER II consortium projects that are focused on cancer.

Abbreviations: CCDI, Childhood cancer data initiative; CSER, Clinical sequencing evidence-generating research (CSER) Consortium; eMERGE, Electronic medical records and genomics (eMERGE) network; MyPART, My pediatric and adult rare tumors network; NCI CORP, National cancer institute community oncology research program; NCI CONNECT, NCI comprehensive oncology network evaluating rare central nervous system tumors; PE-CGS, Participant engagement and cancer genome sequencing (PE-CGS) network.

The six programs not funded by the Cancer Moonshot are broader in their disease focuses, recruiting patients with many different cancer types. For example, the Clinical Sequencing Evidence-Generating Research (CSER) Consortium includes a project that is studying a range of hereditary cancer in adults among diverse populations. The All of Us Research Program is unique among the programs included in this scan because it does not focus specifically on cancer, but rather collects data from any adults, including healthy adults or those with any disease. Five of the six programs not funded by the Cancer Moonshot focus on enrolling underrepresented or medically underserved populations.

### 3.1 Definitions of engagement

Four research programs explicitly defined engagement (Table 2). Key themes reflected the idea that engagement should be: 1) interpersonal, 2) reciprocal, and 3) continuous. The interpersonal theme appears in all four definitions and is reflected in the use of terms such as “trusting” (Casas-Silva et al., 2020), “relationship”(Casas-Silva et al., 2020), and “authentic partnership” (Lemke et al., 2010) between study participants, communities, and researchers. Reciprocity is captured in three of the four definitions with terms such as “mutual respect”(Lemke et al., 2010), “mutually vested” (Casas-Silva et al., 2020), “mutually beneficial” (National Institutes of Health, 2020) and “bi-directional” (National Institutes of Health, 2020) interactions. The continuous theme related to “retaining” (Khodyakov et al., 2018b) participants and “ongoing” (Casas-Silva et al., 2020) interactions throughout “all phases” (National Institutes of Health, 2020) of the research process. However, the definitions focused on different phases of the research process with only one focused on all phases of the research process, including the design, conduct, and uptake of research (National Institutes of Health, 2020). It was most common to focus on the continuous nature of interactions with patients who are study participants.

**TABLE 2:** Written definitions of engagement from research programs that explicitly presented it.

Program	Definition of engagement
Cancer Moonshot Biobank	“The establishment of an ongoing trusting and mutually vested relationship between study participants, healthcare providers and the Biobank” (Casas-Silva et al., 2020).
PE-CGS Network RFA	“An ongoing, bi-directional and mutually beneficial interaction between participants, their communities, and researchers, where participants are included as an integral part of all phases of the research process: including the identification of research priorities and the design, conduct, and uptake of research”(National Institutes of Health, 2020).
All of Us	“The concept of engagement in the [All of Us Research Program] is about partnering with different stakeholders for the purposes of making potential participants aware of the [All of Us Research Program], enrolling them to participate, and retaining them within the program”(Khodyakov et al., 2018b).
eMERGE Network	“A process of inclusive participation that supports mutual respect of values, strategies, and actions for authentic partnership of people affiliated with or self-identified by geographic proximity, special interest, or similar situations to address issues affecting the well- being of the community of focus” (Lemke et al., 2010).

### 3.2 Strategies to engage study participants

**FIGURE 1:**
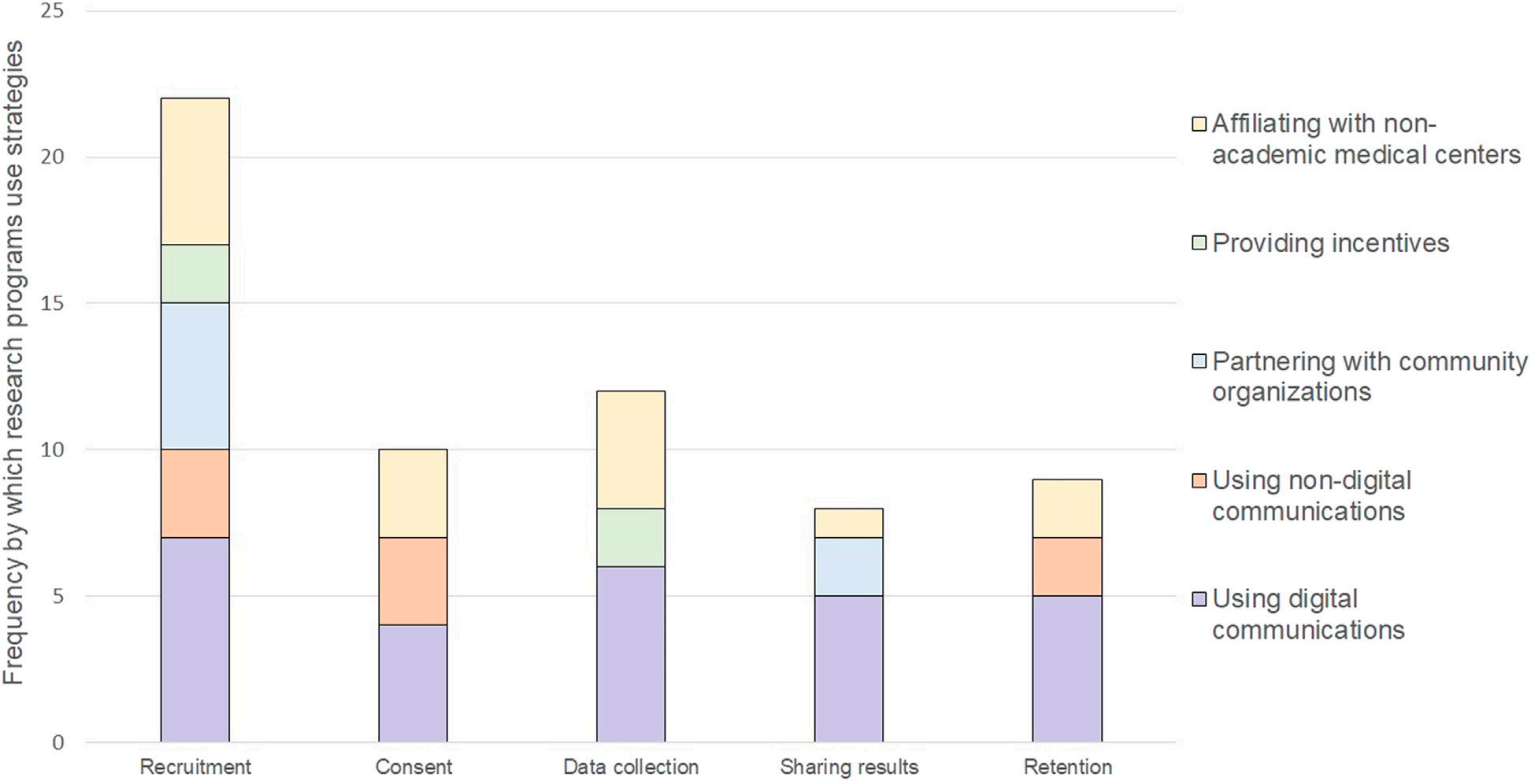
Frequency of strategies used to engage participants throughout conduct of research.

Each research program carried out different study participant engagement strategies to meet enrollment goals, align with their conceptualization of engagement (if described), and overcome known barriers to participation in cancer genomics research. Every research program described opportunities for patients to be involved in the design of their program and its research, including the use of advisory boards, community champions, townhall meetings, deliberative democracy, one-on-one feedback sessions; and human centered-design to guide the strategies they use to engage study participants (Lemke et al., 2010; McCarty et al., 2011; Khodyakov et al., 2018a; Casas-Silva et al., 2020; All of Us Research Program, 2021; O’Daniel et al., 2022). We identified five categories of strategies that programs use to engage patients as study participants. These categories included: 1) using digital communications, 2) using non-digital communications, 3) partnering with community organizations, 4) providing incentives, and 5) affiliating with non-academic medical organizations. We mapped these categories of engagement strategies to different opportunities for engaging study participants during the conduct of research (Figure 1). The strategies are described in more detail below along with brief case examples to describe how and why a strategy was used.

#### 3.2.1 Using digital communications

Using digital communications were relevant to multiple points of engagement with study participants and was the most frequently used strategy. Digital communications, such as websites, videos, electronic newsletters, or social media, offer the opportunity to reach large numbers of prospective participants across broad geographic locations and can enable patients to participate remotely. The programs used digital communications to share information about cancer, genomics, and cancer research; visually depict the steps entailed with participating and explain how long each step should take; and help participants enroll in their study, complete electronic consents, share electronic health records, complete health surveys, and receive individual-level results and aggregate-level study findings. As an example, the Cancer Moonshot Biobank uses its website to support recruitment, post study and research updates, and provide access to a secure patient portal for participants’ receipt of individual biomarker reports (Casas-Silva et al., 2020; Casas-Silva et al., 2021). Some programs tailored their digital communications to reach diverse racial and ethnic populations, including by providing a Spanish version of the website (All of Us Research Program, 2022; Count Me In, 2022; National Cancer Institute, 2022f; National Cancer Institute, 2022a), including images that reflect a diversity of demographics (All of Us Research Program, 2022; Count Me In, 2022; National Cancer Institute, 2022f; National Cancer Institute, 2022a; National Cancer Institute, 2022e; National Cancer Institute, 2022g), and explicitly stating their commitment to promoting diversity and inclusion in research (CSER, 2022b; CSER, 2022a). The use of Frequently Asked Question (FAQs) and infographics were also frequently used to communicate what participation entails (All of Us Research Program, 2022; Count Me In, 2022; National Cancer Institute, 2022a; National Cancer Institute, 2022f; National Cancer Institute, 2022g). Even though digital communications were components of research programs’ study protocols before the COVID-19 pandemic emerged, the pandemic accelerated programs use of it.

#### 3.2.2 Using non-digital communications

Digital communications were reported in the literature and by key informants as being insufficient to promote recruitment or sustain high levels of retention. Non-digital communications were viewed as supporting the needs of those with limited access to technology as well as those who may be less comfortable using it. Non-digital communications were used most commonly to promote recruitment, obtain consent, and promote retention. For recruitment, programs reported using print brochures, radio advertisements, community events, and press coverage (All of Us Research Program, 2021). Their non-digital communications to support retention included support call centers and other strategies successfully implemented by other long-term cohort studies such as reminder calls to participants to keep them engaged in the project; birthday cards to participants from the program; outreach telephone calls to a participant-designated friend or family member (if consented to contact); referral cards for family and friends; and enrollment certificates for recognition (All of Us Research Program, 2021). Print newsletters were also reported to support retention and sharing of overall results. To reach Spanish speaking populations, the studies in the CSER Consortium developed non-digital educational content, informational brochures, and informed consent documents in Spanish (Amendola et al., 2018; CSER, 2022b).

#### 3.2.3 Partnering with community organizations

Partnering with community organizations was viewed as providing a bridge between patients and researchers. Community organizations included patient advocacy organizations, community outreach organizations, and social change organizations. Programs described establishing collaborative relationships with community organizations to facilitate dissemination of information and materials to their respective communities and to serve as cultural brokers who could advocate for the needs and concerns of their community as well as build bridges between their communities and researchers. In some cases, community organizations were also viewed as partners who could support enrollment in programs’ studies. As such, community organizations were valuable partners in promoting recruitment as well as in disseminating aggregate study findings to the community. The MyPART Network, for example, partners with non-profit organizations to work together in numerous ways, including to disseminate research opportunities and findings to relevant audiences and to ensure that the research is centered on the needs of people with rare tumors (National Cancer Institute, 2022e). Additionally, the PE-CGS Network identified partnerships with patient-centered advocacy and community organizations as an important way to engage patients, optimize recruitment, and seamlessly return results (National Cancer Institute, 2022g). Moreover, these partnerships were seen as a vital way to gather input and feedback from those affected by the research, including patients and their families and communities (National Cancer Institute, 2022g).

#### 3.2.4 Providing incentives

Extrinsic incentives were described as a way to demonstrate respect for study participation. They were also viewed as a mechanism to help address barriers to participation, such as competing demands for time or limited access to transportation. They often took the form of direct payment, gift cards, and/or parking validation. For example, in the Connect for Cancer Prevention Study, after participants complete their first survey and donate their first blood sample, they will receive $25 in cash or as a gift card (depending on the healthcare system they’re affiliated with) (National Cancer Institute, 2022d). The study also covers parking validation when donating samples in person (National Cancer Institute, 2022d). In some instances incentives also included providing access to experts who often have more experience with particular types of cancer than patients’ personal medical team and may be able to provide advice on cancer treatments and/or identify relevant treatment trials. As part of NCI-CONNECT, for instance, study participants can visit with experts associated with study’s neuro-oncology care team for an evaluation and consultation (National Cancer Institute, 2022f). The consultation is free and provides participants with accurate diagnosis and treatment guidance. Generally, incentives were considered as one way to support the participation of traditionally underrepresented populations and, in a small way, rectify past negative and harmful experiences with research (All of Us Research Program, 2021).

#### 3.2.5 Affiliating with non-academic medical centers

Affiliating with non-academic medical centers was considered an opportunity to reach a larger and more diverse patient population treated in a variety of healthcare delivery settings. Programs indicated that affiliating with non-academic medical centers could accelerate accrual, facilitate the collection of data and biospecimens, increase the diversity of participants, and enhance the relevance of study findings. Non-academic medical centers included regional medical centers, federally qualified health centers, the Veterans Health Administration, integrated healthcare systems, and institutions affiliated with the NCI Community Oncology Research Program (NCORP) (All of Us Research Program, 2021; National Cancer Institute, 2022a; National Cancer Institute, 2022d). As one example, the Cancer Moonshot Biobank aims to collect and distribute longitudinal cancer biospecimens from study participants receiving standard of care therapy at participating NCI Community Oncology Research Program (NCORP) institutions (The International Society for Biological and Environmental Repositories, 2022). Affiliating with NCORP sites and making funding available for them to implement local engagement strategies is designed to support the goal of increasing participation among rural and other medically underserved communities as well as enrolling participants who represent the racial and ethnic diversity of the United States (The International Society for Biological and Environmental Repositories, 2022).

### 3.3 Questions about current state of patient engagement in cancer genomics research

Key informants raised a number of questions about the current state of patient engagement in cancer genomics research that fell into three broad categories: 1) what does patient engagement entail? 2) what are the barriers to implementing engagement strategies? and 3) how do we know if the engagement strategies are working? These categories are summarized in Table 3 and accompanied by representative quotations from our key informants.

**TABLE 3:** Overarching questions about current state of engagement.

Overarching question	Representative quote
What does patient engagement entail?	“We talk a lot about what does engagement mean to a patient who participates in research. What are they looking for and what do they want from participating in a research study? What are their needs?”
	“You know participant engagement is not the same as participant recruitment and retention. Those are separate. You can, in engagement of community, ask how to recruit and how to retain.”
	“Many researchers think that patient engagement means increasing recruitment into clinical trials period, end of story. Obviously, that’s a big part of it, but there’s no way that should be all of it.”
What are the barriers to implementing engagement strategies?	“It’s still a real challenge to reach people who are not able to connect with our resources. It could be due to literacy, access to technology, or even just not having the time to do so.”
	“Projects take for granted that diverse research participation is going to result in diverse benefit to diverse populations. We do a disservice to our research participants by implying that they should take this for granted.”
	“[Remote] enrollment is more transactional. You’re not really getting to know anybody. You say sign this form online, fill out this questionnaire, send your sample. But in other situations, you must be relational and build trust first.”
How do we know if the engagement strategies are working?	“It’s really that we need to understand what engagement produces … and really trying to produce social change that is needed for precision medicine and public health. But we do not yet know the impact of engagement.”
	“These are contributions from living humans who really care deeply about the research that is going on because of their participation, but we still have not figured out if our digital projects, products, and services are ensuring that they have good experiences and that we are meeting their needs.”
	“Engagement strategies are really at the front end, but we’re going to encounter a whole other set of issues when we return information folks in the study to people. Would not it be good to know how that’s landing?”

The questions raised about what patient engagement entails highlighted discordance about the relationship between engagement and recruitment. The comments revealed that many activities used for engagement may also be used for recruitment and figuring out how to separate these ideas is difficult. For some key informants’ the term patient engagement should only be used to refer to patients’ informing the design and conduct of research, but not activities for patients as study participants, in spite of current practice. Additionally, among key informants who applied the term patient engagement to patients in their role as study participants, they explicitly wondered what patients were seeking from their participation in research.

Related to the questions about the barriers to implementing engagement strategies, there were concerns about digital communication’s ability to overcome technological barriers to participation as well as barriers related to distrust. There was doubt that digital communications alone are sufficient for developing trust and mutually respectful relationships, which in line with the definitions of engagement, were perceived to be crucial components of engaging diverse study participants. Building relationships with patients’ and their communities before asking them to participate in a study was an important consideration. One concern was whether researchers are misrepresenting the benefits of study participation, and the inadvertent consequences of over-promising and under-delivering on the benefits of participating in genomics research.

In terms of questions about how do we know if engagement is working, key informants noted the importance of studying the effectiveness of patient engagement from the short-term to the long-term. In the short-term key informants expressed questions about process measures such as how well programs were meeting study participants’ expectations and needs. In the long-term, key informants wanted evidence on which engagement strategies are meeting program goals of reaching diverse participants and how engagement strategies are achieving health outcomes such as transforming cancer therapy and cancer care delivery. They noted, however, that such evidence is lacking at this point.

## 4 Discussion

Patient engagement in cancer genomics research is supported by national priorities such as the Cancer Moonshot to reflect the full diversity of the United States (National Cancer Institute, 2016; The All of Us Research Program Investigators, 2019). The findings of this environmental scan demonstrate the intent to increase the representation of diverse populations in these ten cancer genomics research programs. They strive to include study participants who represent broad and diverse populations, including across age, race, ethnic group, geographic location, and types of cancers. Additionally, they aim to increase study participation among populations with understudied cancers and those historically exploited or excluded in cancer genomics research. As highlighted by the goals of the Cancer Moonshot, the representation of diverse populations in cancer research is critical for rapidly advancing our understanding of cancer and improving patient care.

The meaning of engaging patients as study participants was generally broad. We cataloged research programs existing written definitions of engagement in cancer genomics research, and in doing so, identified a shift in the meaning of patient engagement. In particular, as reported in Table 2, we found that engagement of patients as study participants includes being interpersonal and reciprocal on an ongoing basis, which is more expansive than categorizing study participation as passive engagement. This understanding of engagement contradicts a historical emphasis of engagement on recruitment. In support of engagement as an ongoing process, we identified that patient engagement in cancer genomics research entails interactions between participants and researchers across five stages of conducting a study—recruitment, consent, data collection, return of results, and retention—using five types of strategies. The most prominent of these strategies was digital communications, which was the only strategy used across all five stages of conducting a study.

Through this review we found three existing needs to meaningfully advance the science of patient engagement in cancer genomics research and serve as a baseline from which we can track our progress on addressing those needs. The three needs are to: 1) reach an agreement on the meaning of patient engagement; 2) develop a clear taxonomy of measures to be able to assess the quality and comparative effectiveness of engagement strategies; and 3) identify the comparative effectiveness of engagement strategies. Addressing these three needs would facilitate the use of engagement strategies in cancer genomics research and would enable a better understanding of how to tailor different engagement strategies to different groups.

First, the field of cancer genomics research needs to reach an agreement on the meaning of patient engagement because ambiguity remains. While the spirit of the definition of engagement was arguably similar, only four of the ten programs had an explicit, written definition of engagement as shown in Table 2. Of these definitions, three of the four referred to patient engagement as occurring, at least in part, between researchers and patients as study participants from recruitment to retention through ongoing, reciprocal relationships. It is not clear if the remaining programs would have defined engagement in concordant ways because key informants raised conflicting viewpoints about the meaning of engagement. As indicated in Table 3, some key informants were adamant that engagement is different from recruitment and others continued to think of recruitment as an important part of engagement. Regardless, in synthesizing the written definitions from Table 2, we observe that the emphasis on engaging patients as study participants in ongoing, reciprocal relationships expands upon previous conceptualizations; namely, where engaging study participants was classified as having a passive level of involvement (Domecq et al., 2014; Shippee et al., 2015). While a broad sense of engagement might enable reaching specific communities, developing a consistent definition would address key questions about the purpose and priorities of patient engagement as identified by the key informants and would also help operationalize and assess patient engagement efforts. Recent work synthesizes definitions of engagement across diverse academic disciplines (Schuster et al., 2022a), including engagement marketing (Lewis et al., 2022), and could be a foundation for operationally defining engagement in cancer genomics research.

Second, the field ought to develop a clear taxonomy of engagement measures to assess the comparative effectiveness of patient engagement strategies in cancer genomics research. Currently, there is a lack of available measures and measures that could be appropriate for different populations. The purpose of an evaluation informs the metrics to be used. The taxonomy could be grounded in a Donabedian model that classifies measures of engagement according to structure, process, and outcomes. The type of measure would inform the methods—quantitative, qualitative or mixed-methods—needed to assess them as well as the necessity of demonstrating appropriate instrument development and testing (e.g., validity, reliability, feasibility, acceptability, responsiveness, interpretability, appropriateness, precision). Developing a taxonomy of engagement measures would support clear reporting of patient engagement so that researchers could replicate similar approaches and also fulfill study reporting guidelines known as the ‘Guidance for Reporting Involvement of Patients and the Public (GRIPP2) (Staniszewska et al., 2017). The development of such a taxonomy could address several questions raised by the key informants. For instance, a taxonomy could ensure that measures exist to understand what patients perceive as transactional. It could also ensure that measures exist to appropriately assess engagement across all five stages of conducting a study–recruitment, consent, data collection, return of results, and retention–not just at the start of the study.

Third, the field lacks empirical research testing and comparing the effectiveness of engagement strategies in cancer genomics research. At the present time, it is not possible to recommend best practices on which of the engagement strategies are optimal for any given study activity. Key informants indicated the importance of understanding the value of engagement, and not overstating the assumed value of it. Over-promising the benefits of research participation to diverse populations could have the downstream effect of eroding trust and ongoing, long-term engagement. As such studying engagement strategies is a key need for future research. Some literature suggests the importance of studying engagement through the lens of adaptive learning systems that are initially informed by existing strategies and enabled to be carried out through iterative effectiveness testing (All of Us Research Program, 2021). With this perspective, and given that challenges will inevitably arise, there is a need for programs to be able to nimbly make modifications to their study protocols and materials. Committing to research that empirically tests the comparative effectiveness of engagement strategies raises a potentially difficult task. Researchers, funders, and journals will need to be willing to report and publish on failed efforts. This will be particularly important if research programs are going to in fact design and implement strategies that effectively support the inclusion of all participants.

This research is limited in several ways. First, we only briefly address the role of patient engagement in the research programs during the preparatory phase of research, as defined previously (Domecq et al., 2014; Shippee et al., 2015), and we did not address its role during the translational phases of research. These are, however, important aspects of patient engagement where engagement could inform developing research questions, setting priorities, developing protocols, guiding enrollment, analyzing data, and disseminating key findings. There are many strategies for this type of engagement and evidence of their use came up during our scan. However, this was beyond the scope of this environmental scan and future research should assess programs’ use of patient engagement across these additional phases of research. Another major limitation is that we cannot be sure we captured all cancer genetic research programs that are doing engagement. Similarly, we cannot be sure that we captured all types of engagement strategies being used by the research programs nor could we determine the motives driving use of the different strategies. While environmental scans focus on the phenomenon being studied, this highlights a key opportunity for deeper qualitative research to understand the motivations for patient engagement and to compare how engagement is being done and measured within and across research programs (Schuster et al., 2022b). That said, our environmental scan was thorough and was meant to represent a snapshot of the time in which we reviewed them. Additionally, the content we report in was verified as of finalizing the manuscript, so findings are as up to date as possible. Finally, we did not directly engage patients as a data source in this environmental scan because we sought to characterize programs’ current definitions and practices of engagement. Future work is still needed because the involvement and opinions of patients are critical to advancing the field of engagement in cancer genomics research and several research programs noted how they strive to embed patients throughout their work.

The study provides a contemporary look at patient engagement in the context of research programs studying cancer genomics in the US. We utilized a comprehensive environmental scan to select and characterize the evidence. The findings signal a deep commitment to increasing research participation among diverse populations and a conceptualization of engagement that is responsive to overcoming barriers to participation. Yet, the field has clear needs to inform its practice of patient engagement, which entails advancing the science of patient engagement in cancer genomics research. The field must define what engagement entails, identify how to measure engagement, and generate evidence on the effectiveness of engagement strategies. Addressing these needs could enable patient engagement to fulfill its potential and accelerate the pace of cancer genomic discoveries.

## Data Availability

The original contributions presented in the study are included in the article/Supplementary Material, further inquiries can be directed to the corresponding author.
