# Supplementary material for: Promoting patient engagement in cancer genomics research programs: An environmental scan
Source: Front Genet. 2023 Jan 18;14:1053613. doi: 10.3389/fgene.2023.1053613 (PMC9889863; doi:10.3389/fgene.2023.1053613)
Supplement: Supplementary file 1 [file Table1.DOC]

***Supplementary Material***

**1 Literature review: Search terms**

**Google Scholar**: Limited to 2010-2021

allintitle: ("patient–researcher" OR "patient engagement" OR "patient-partnered" OR "community engagement" OR "community participation" OR "community partnership" OR “community based participatory research”) (cancer OR genetic OR genomic)

**Web of Science:**

AB= (("Genom*" OR "Exome*" OR "Genetic*" OR "rare gene*" OR "Precision medicine" OR "Personalized medicine" OR "Personalised medicine" OR "Individualized Medicine" OR "Predictive Medicine" OR "High-Throughput Nucleotide Sequencing" OR "Next generation sequencing" OR "Genetic Polymorphism*" OR "Polymorphism*" OR "Polygenic" OR "Pharmacogenetic*" OR "Pharmacogenomic*" OR "massive parallel sequencing" OR "whole genome sequencing") AND ("Neoplas*" OR "Maligna*" OR "Cancer*" OR "Rare Cancer*" OR "Carcinoma*" OR "Tumor*" OR "Tumour*" OR "Medical Oncology") AND ("Patient Participation" OR "Patient Involvement" OR "Patient Empowerment" OR "Patient Activation" OR "Community Participation" OR "Community Involvement*" OR "Consumer Participation" OR "Consumer Involvement" OR "Public Participation" OR "Community Action*" OR "Community Based Participatory Research" OR "academic-community partnership" OR "Patient Preference*" OR "Patient-Partnered Research" OR "Patient Collaboration" OR "Researcher-Subject Relations*" OR "Professional Patient Relations*" OR "Professional-Patient Relations*" OR "Stakeholder Participation" OR "Stakeholder Engagement") ) OR TI=(("Genom*" OR "Exome*" OR "Genetic*" OR "rare gene*" OR "Precision medicine" OR "Personalized medicine" OR "Personalised medicine" OR "Individualized Medicine" OR "Predictive Medicine" OR "High-Throughput Nucleotide Sequencing" OR "Next generation sequencing" OR "Genetic Polymorphism*" OR "Polymorphism*" OR "Polygenic" OR "Pharmacogenetic*" OR "Pharmacogenomic*" OR "massive parallel sequencing" OR "whole genome sequencing") AND ("Neoplas*" OR "Maligna*" OR "Cancer*" OR "Rare Cancer*" OR "Carcinoma*" OR "Tumor*" OR "Tumour*" OR "Medical Oncology") AND ("Patient Participation" OR "Patient Involvement" OR "Patient Empowerment" OR "Patient Activation" OR "Community Participation" OR "Community Involvement*" OR "Consumer Participation" OR "Consumer Involvement" OR "Public Participation" OR "Community Action*" OR "Community Based Participatory Research" OR "academic-community partnership" OR "Patient Preference*" OR "Patient-Partnered Research" OR "Patient Collaboration" OR "Researcher-Subject Relations*" OR "Professional Patient Relations*" OR "Professional-Patient Relations*" OR "Stakeholder Participation" OR "Engagement") )

**PubMed:** Limited to 2010-2021

("genomics"[MeSH] OR "genome"[Text Word] OR "exome"[MeSH] OR "exome"[Text Word] OR "genetic"[Text Word] OR "rare-gene"[Text Word] OR “rare-cancer”[Text Word] OR “Sequencing"[Text Word] OR "High-Throughput Nucleotide Sequencing"[MeSH] OR "whole genome sequencing"[MeSH] OR "polymorphism"[Text Word] OR "Polygenic"[Text Word] OR "pharmacogenetic"[Text Word] OR "pharmacogenomic"[Text Word]) AND ("Pharmacogenetics”[MeSH] OR "neoplasm"[Title/Abstract] OR "malignancy"[Title/Abstract] OR "cancer"[Title/Abstract] OR "carcinoma"[Title/Abstract] OR "Carcinoma"[MeSH] OR "tumor"[Title/Abstract] OR "tumour"[Title/Abstract] OR "Oncology"[Title/Abstract] OR “Medical Oncology”[MeSH]) AND ("Patient-Participation"[MeSH] OR "Patient-Involvement"[Title/Abstract] OR "Patient-Empowerment"[Title/Abstract] OR "Patient-Activation"[Title/Abstract] OR "Community-Participation"[Title/Abstract] OR "Community Participation"[MeSH] OR "community-involvement"[Title/Abstract] OR "Consumer-Participation"[Title/Abstract] OR "Consumer-Involvement"[Title/Abstract] OR "Public-Participation"[Title/Abstract] OR "community-action"[Title/Abstract] OR "Community-Based-Participatory-Research"[Title/Abstract] OR "Community Based Participatory Research"[MeSH] OR "academic-community-partnership"[Title/Abstract] OR "patient-preference"[Title/Abstract] OR "Patient Preference"[MeSH] OR "Patient-Partnered"[Title/Abstract] OR "Patient-Collaboration"[Title/Abstract] OR "researcher-subject-relationship"[Title/Abstract] OR "Researcher-Subject Relations"[MeSH] OR "professional-patient-relationship"[Title/Abstract] OR "professional-patient-relationship"[Title/Abstract] OR "Professional Patient Relations"[MeSH] OR "Stakeholder-Participation"[Title/Abstract] OR “Stakeholder Participation”[MeSH] OR "Stakeholder-Engagement"[Title/Abstract] OR "patient-engagement"[Title/Abstract] OR "patient-researcher"[Title/Abstract] OR "community-engagement"[Title/Abstract] OR “stakeholder-engagement”[ Title/Abstract] OR "Healthcare Disparities"[MAJR])

**Embase**:

('genom*':ab,ti OR 'exome*':ab,ti OR 'genetic*':ab,ti OR 'rare gene*':ab,ti OR 'precision medicine':ab,ti OR 'personalized medicine':ab,ti OR 'personalised medicine':ab,ti OR 'individualized medicine':ab,ti OR 'predictive medicine':ab,ti OR 'high-throughput nucleotide sequencing':ab,ti OR 'next generation sequencing':ab,ti OR 'genetic polymorphism*':ab,ti OR 'polymorphism*':ab,ti OR 'polygenic':ab,ti OR 'pharmacogenetic*':ab,ti OR 'pharmacogenomic*':ab,ti OR 'massive parallel sequencing':ab,ti OR 'whole genome sequencing':ab,ti) AND ('neoplas*':ab,ti OR 'maligna*':ab,ti OR 'cancer*':ab,ti OR 'rare cancer*':ab,ti OR 'carcinoma*':ab,ti OR 'tumor*':ab,ti OR 'tumour*':ab,ti OR 'medical oncology':ab,ti) AND ('patient participation':ab,ti OR 'patient involvement':ab,ti OR 'patient empowerment':ab,ti OR 'patient activation':ab,ti OR 'community participation':ab,ti OR 'community involvement*':ab,ti OR 'consumer participation':ab,ti OR 'consumer involvement':ab,ti OR 'public participation':ab,ti OR 'community action*':ab,ti OR 'community based participatory research':ab,ti OR 'academic-community partnership':ab,ti OR 'patient preference*':ab,ti OR 'patient-partnered research':ab,ti OR 'patient collaboration':ab,ti OR 'researcher-subject relations*':ab,ti OR 'professional patient relations*':ab,ti OR 'professional-patient relations*':ab,ti OR 'stakeholder participation':ab,ti OR 'stakeholder engagement':ab,ti)

**2 Literature review flow diagram**

Full texts assessed for eligibility
(n = 138)

Records included
(n = 13)

Excluded based on title and abstract

(n = 751)

Titles and abstracts screened

(n = 905)

Duplicates removed
(n = 218)

**3 Semi-structured interview guide with key informants**

1. Can you tell me about your background and current role with your organization?
2. We are trying to understand how different stakeholders think about participant engagement. How you would define participant engagement?

**Prompt:** Do you think there are differences between participant engagement generally and participant engagement in the context of cancer genomics?

**Prompt:** Can you tell me about any frameworks/articles that provide seminal information about participant engagement in cancer genomics?

**Prompt:** Do you find information about participant engagement?

1. Can you describe for the me different stages of cancer genomics?
2. We would like to learn about strategies and activities for participant engagement. In your current role, how are you involved in any participant engagement initiatives related to cancer genomics?

**Prompt:** Can you tell me about the participants that are engaged in these activities?

**Prompt:** At what stage in their cancer journey are they being engaged?

**Prompt:** Do you tailor participant engagement activities to different groups of patients?

1. What do you think works well with these initiatives, and what could be improved?
2. Are there any approaches you know of that are used to evaluate the success of participant engagement generally? In cancer genomics specifically?
3. Are you aware of or do you use any resources of guidance to help you plan and execute patient engagement activities? Can you please describe these?
4. Outside of your work, do you know of other strategies or activities for engaging participants in cancer genomics?
5. Is there anything about participation engagement in cancer genomics that we did not discuss but which you would like me to know?
6. Do you have recommendations for any other professionals we should talk to learn more about participant engagement in cancer genomics?
